# Resistance to ursodeoxycholic acid-induced growth arrest can also result in resistance to deoxycholic acid-induced apoptosis and increased tumorgenicity

**DOI:** 10.1186/1471-2407-6-219

**Published:** 2006-09-01

**Authors:** Ashley A Powell, Sandeep Akare, Wenqing Qi, Pascal Herzer, Samira Jean-Louis, Rebecca A Feldman, Jesse D Martinez

**Affiliations:** 1 Cancer Biology Interdisciplinary Program, University of Arizona, Arizona Cancer Center, Tucson, AZ, 85724, USA; 2 Department of Surgery, Stanford University, MSLS P229, 1201 Welch Road, Stanford, CA 94305, USA; 3 Department of Cell Biology and Anatomy, University of Arizona, Arizona Cancer Center, Tucson, AZ, 85724, USA; 4 Department of Pathobiology, College of Veterinary Medicine University of Illinois Urbana Champagne, 2001 South Lincoln Avenue, Urbana, IL 61802, USA; 5 Department of Medicine, University of Arizona, Arizona Cancer Center, Tucson, AZ, 85724, USA; 6 Applied Biosciences Program, University of Arizona, Tucson, AZ 85724, USA; 7 The Scripps Research Institute, Office of Technology Development, 10550 N. Torrey Pines Rd., La Jolla, CA 92037, USA; 8 Department of Nutritional Sciences, University of Arizona, Tucson AZ 85724, USA

## Abstract

**Background:**

There is a large body of evidence which suggests that bile acids increase the risk of colon cancer and act as tumor promoters, however, the mechanism(s) of bile acids mediated tumorigenesis is not clear. Previously we showed that deoxycholic acid (DCA), a tumorogenic bile acid, and ursodeoxycholic acid (UDCA), a putative chemopreventive agent, exhibited distinct biological effects, yet appeared to act on some of the same signaling molecules. The present study was carried out to determine whether there is overlap in signaling pathways activated by tumorogenic bile acid DCA and chemopreventive bile acid UDCA.

**Methods:**

To determine whether there was an overlap in activation of signaling pathways by DCA and UDCA, we mutagenized HCT116 cells and then isolated cell lines resistant to UDCA induced growth arrest. These lines were then tested for their response to DCA induced apoptosis.

**Results:**

We found that a majority of the cell lines resistant to UDCA-induced growth arrest were also resistant to DCA-induced apoptosis, implying an overlap in DCA and UDCA mediated signaling. Moreover, the cell lines which were the most resistant to DCA-induced apoptosis also exhibited a greater capacity for anchorage independent growth.

**Conclusion:**

We conclude that UDCA and DCA have overlapping signaling activities and that disregulation of these pathways can lead to a more advanced neoplastic phenotype.

## Background

Bile acids are polar derivatives of cholesterol which are synthesized in the liver and stored in the gall bladder [1]. During digestion bile is excreted into the intestinal tract where bile acids aid in the absorption of dietary fats. Although the majority of the bile acids is reabsorbed and reused a small fraction (1–4%) is not reabsorbed and passes into the colon [2]. Here the primary bile acids, those bile acids that are produced in the liver, are modified by enteric bacteria dehydroxylating the cholesterol core and removing the conjugated amino acid to produce unconjugated secondary bile acids. These secondary bile acids, principally deoxycholic acid, have been associated with increased risk for colon cancer

Epidemiological and animal model studies support the concept that bile acids may play a role in the development of colon cancer. Studies of populations that eat high fat diets which promote more bile acid production show increased risk for colon cancer [3,4] and patients diagnosed with colon cancer have elevated levels of serum bile acids, especially deoxycholic acid (DCA) [3,5]. In studies using animal models DCA was found to act synergistically with carcinogens to increase colon tumorigenesis [6,7] and could cause transformation of cells in vitro [8]. Collectively these observations suggest that DCA may be a tumor promoter. However it should be noted that not all bile acids act to promote colon tumor development. Ursodeoxycholic acid (UDCA) suppresses the development of colon tumors in AOM-treated rats [9,10] and two studies in human subjects suggest that UDCA can reduce the risk of developing colorectal cancer [11-13]. Hence, in spite of having very similar chemical structures, these two bile acids have very distinct biological activities both at the organismal level as well as in vitro [14]. To date the mechanism that accounts for this difference in function is not clear.

The mechanism through which bile acids bring about there biological effects is not well understood, however, there is a growing body of evidence indicating that bile acids can regulate gene expression [15-18]. DCA has been shown to activate a number of mitogenic and apoptosis associated signaling pathways which is consonant with its proposed tumor promoting abilities including the epidermal growth factor receptor and the raf/mek/erk pathway [19-21], protein kinase C [22-24], the AP-1 transcription factor [25-27], and Cox2 [17] all of which are known to be dysregulated during colon tumorigenesis. Much less is known about the signaling mechanisms activated by UDCA. However, in general UDCA displays activities that are in opposition to those exhibited by DCA. For instance UDCA can suppress activation of ras, EGFR-raf/mek/erk pathway and AP-1 [19] and is cytoprotective as opposed to cytotoxic DCA [28,29]. Similarly, while DCA interferes with functioning of the p53 tumor suppressor, UDCA does not [21]. Interestingly, we found that bile acids are not readily taken up by colonic cells [30], but instead initiate intracellular signaling by their action at cell membrane [22] in ligand independent manner possibly through specialized domains like caveolae [31]. Given the mode of action of DCA and UDCA on cell membranes, it is likely that these two bile acids can act on some of the same signaling pathways.

Understanding if DCA and UDCA utilize same pathways to bring about diametrically opposed biological outcome is very important in characterizing the role bile acids play in tumorigenesis. In addition, before therapeutically targeting DCA activated signaling to overcome the DCA mediated colon carcinogenesis, it is important to address whether UDCA employs similar signaling pathways as DCA. In order to gain insight into overlap in signaling pathways activated by DCA and UDCA, we isolated cell lines resistant to UDCA induced growth arrest and then tested these for their response to DCA induced apoptosis, since, the most significant biological effects for DCA and UDCA have been shown to be apoptosis and growth arrest, respectively. Characterization of these resistant cells demonstrated that some were also cross resistance to the effects of DCA suggesting that DCA and UDCA signaling activity may overlap. Importantly, we found evidence that resistance to some DCA-activated signaling lead to a more neoplastic phenotype. The relevance of these finding to colon cancer are discussed.

## Methods

### Reagents

DCA, cholic acid, and hyoDCA were obtained from Sigma Chemical (St. Louis, MO) and UDCA from Calbiochem (La Jolla, CA). All were maintained as 100 mM stock solutions in water. Upon addition of bile acids to media, no change in pH was observed. Etoposide, cisplatin, and adriomyosin were all obtained from Sigma Chemical Co. (St. Louis, MO)

### Cell culture

The HCT116 colon cancer cell line was used as the parental cell line in all experiments and was purchased from the American Type Culture Collection (Rockville, Maryland). All cell lines were propagated at 37°C and 5% CO_2 _in a humidified atmosphere in Dulbecco's modified Eagle's medium (DMEM) (Gibco/BRL, Gaithersburg/MD) supplemented with 10% fetal bovine serum (Gibco/BRL), 100 units of penicillin, 100 mg of streptomycin, 2 mM L-glutamine, 4 mM sodium pyruvate and 100 μM non-essential amino acids.

### Derivation of UDCA resistant cell lines

Parental HCT116 cells were plated onto a 162 cm^2 ^flask with 50 milliliters of fresh DMEM and allowed to attach and grow for 24 hours. This produced a cell monolayer that was approximately 40% confluent. These cells were mutagenized by incubation with ethyl methane sulfonate (Sigma) at a final concentration of 500 μg/ml for 12 hours. Cells were then rinsed three times with DMEM, re-fed with fresh DMEM before returning to the incubator for 24 hours. Cells were then split into 20 ten centimeter dishes and allowed to grow for 24 hours prior to the addition of UDCA to a final concentration of 500 μM in each dish. Cells were refed with fresh DMEM supplemented with 500 μM UDCA once a week for four weeks at which time colonies of UDCA resistant cells appeared. From this treatment 47 UDCA resistant colonies emerged, 41 of which were successfully expanded into peremanent cell lines. Once the lines were expanded into 10 cm dishes, cells were maintained in DMEM supplemented with 250 μM UDCA. These lines were designated HOMUR cells for HCT116 Odd Morphology UDCA resistant.

### Screening HOMUR lines for cross resistance to DCA and hyoDCA

HOMUR cells were plated at 100,000 cells per 35 mm dish and then incubated for 24 hours prior to the addition of either DCA or hyoDCA to a final concentration of 500 μM. Cells exposed to DCA were incubated for 24 hours and then harvested and the fraction of cells undergoing apoptosis determined as described below. HOMUR cells exposed to hyoDCA were incubated for 48 hours with this bile acid and then the fraction of apoptotic cells determined.

### Apoptosis assay

For apoptosis assays 100,000 HCT116 cells were plated onto 60 mm tissue culture plates and allowed to attach for 24 hours. This procedure produced a cell monolayer that was 30–40% confluent at the time bile acids were added. The cells were treated with 500 μM bile acids for the times indicated. The media were removed and saved and the remaining attached cells rinsed in PBS and harvested by trypsinization. The cell pellet was re-suspended in the saved media. The number of apoptotic cells was then quantitated by staining with acridine orange and ethidium bromide as described previously [14].

### Anchorage independent growth

To test for anchorage independent growth cells were grown in 0.6% agarose as follows. A stock of 1.2% LMP agarose (Gibco) was autoclaved and then the solution equilibrated at 37°C for 30 minutes. The LMP agarose was diluted 1:1 with DMEM and one milliliter of the mixture poured into each well of a 6 well plate to form a basal layer. This basal layer was allowed to solidify for 10 minutes at 4°C prior to reequilibrating at room temperature for 30 minutes. The top layer was similar to the basal layer, but contained 5,000 cell per well. The top layer was allowed to solidify at room temperature for approximately 15 minutes and the plates were then transferred to a 37°C incubator with 5% CO_2_. The following day, one milliliter of medium was added to each well, and the cells refed every 3–4 days for 2.5 weeks. Three sets of experiments were performed in triplicate. The total number of colonies was counted and the percent colony formation determined.

### Statistical analysis

Statistical analysis of data was performed using Sigmastat statistical analysis software. In all cases a p value of <0.05 was considered the threshhold for significance.

## Results

### Isolation and characterization of cells resistant to UDCA-induced growth arrest

**Figure 1:**
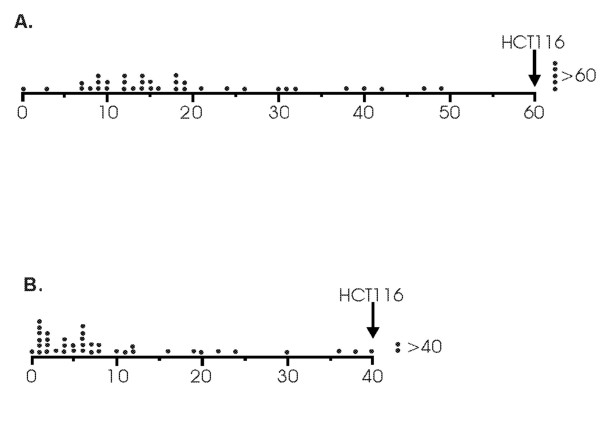
**The majority of HOMUR cell lines exhibit resistance to DCA-induced and hyoDCA-induced apoptosis. **(A) HOMUR cells were incubated with 500 micromolar DCA for 24 hours and the fraction of apoptotic cells determined as described in Methods. The apoptotic value for each of the 41 HOMUR lines is depicted as a dot along a scale extending from 0 to 60% apoptosis. The extent of apoptosis obtained for parental HCT116 cells is indicated with an arrow at the right. Also on the right are dots representing individual HOMUR cell lines with apoptotic values greater than 60%. (B) HOMUR cells were incubated with 500 micromolar hyoDCA for 48 hours and the fraction of apoptotic cells determined as in panel A. Each dot represents a single HOMUR cell line and its placement is representative of the apoptotic value for that cell line. The apoptosis obtained using HCT116 cells is indicated with an arrow at the right. In addition, HOMUR lines with apoptotic values greater than 40% are represented as dots to the right of the graph.

To test for overlap in signaling and elucidate the differences in signaling activated by DCA and UDCA each of the HOMUR cell lines was tested for resistance to apoptosis induced by DCA and hyoDCA as described in the Methods section (Figure 1A). These two bile acids were selected because DCA is known to induce apoptosis, but has no ability to inhibit cell proliferation [14]. In contrast, we found that hyoDCA initially causes growth arrest followed by apoptosis in HCT116 cells (unpublished data). Because hyoDCA exhibits biological activity that is intermediate to DCA and UDCA we also examined the HOMUR lines for resistance to the cytotoxicity of this bile acid. When tested for cross resistance to these other bile acids we found that the majority of HOMUR cell lines demonstrated some resistance to apoptosis induced by either DCA or HyoDCA. When tested for DCA-induced apoptosis 88% (36 out of 41) of the HOMUR lines exhibited reduced apoptosis compared to the 60% apoptosis observed with parental HCT116 cells when treated with DCA under the same conditions (Figure 1A). Indeed apoptosis was virtually undetectable in one HOMUR line (HOMUR 7) when incubated with 500 micromolar DCA for 24 hours. The average percent DCA-induced apoptosis for all of the resistant HOMUR lines was 18.9%, a reduction of ~32% relative to similarly treated parental HCT116 cells. We found that 93% (38 out of 41) of the HOMUR lines also exhibited reduced apoptosis induced by hyoDCA when compared with the parental control values suggesting that HOMUR lines were also resistant to the cytotoxic effects of this bile acid. The average percent hyoDCA-induced apoptosis for the resistant HOMUR lines was 10% a reduction of ~25% relative to parental HCT116 cells. Moreover, the HOMUR 7 cells again showed no evidence of apoptosis when treated with hyoDCA indicating that this line was resistant to apoptosis induced by both bile acids. Interestingly, a small fraction, 12% (5 out of 41) for DCA and 5% (2 out of 41) for hyoDCA, of HOMUR lines showed increased apoptosis than parental HCT116 cells when treated with these bile acids suggesting that the mutations that made them resistant to UDCA made them more sensitive to bile acid-induced apoptosis. From these experiments we concluded that disruption of the pathways that enable UDCA to inhibit cell growth can also lead to resistance to the cytotoxic effects induced by other bile acids.

### Resistant HOMUR cells also show resistance at the biochemical level

**Figure 2:**
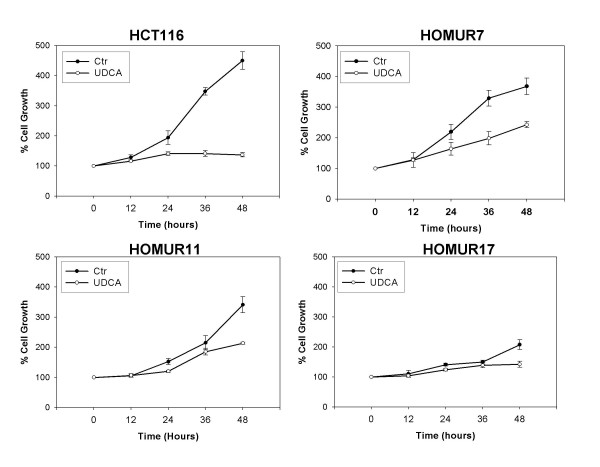
**Selected HOMUR cells show resistance to UDCA-induced growth arrest. **(A) HCT116, (B) HOMUR 7, (C) HOMUR 11, or (D) HOMUR 17 cells were plated into 35 mm plates and allowed to attach for 48 hours. Subsequently the plates were either left untreated (closed circles) or 500 micromolar UDCA was added to the media (open circles) and incubation continued. Sample plates were removed at the time intervals indicated and the number of cells counted. Experiments were performed in triplicate and the average values graphed. Error bars represent standard deviation.

From our bank of HOMUR cells we selected three lines representative for further study. HOMUR7 cells were selected because of their profound resistance to DCA-induced apoptosis. The HOMUR11 line was selected because the bile acid-induced apoptosis for both DCA and hyoDCA was close to the average value for bile acid-induced apoptosis by the group of HOMUR cell lines as a whole. The HOMUR17 line was selected because it was one of a small fraction of HOMUR lines that were more sensitive to bile acid-induced apoptosis than were the parental cells. Hence, HOMUR 7, 11, and 17 represent the spectrum of phenotypes present in our group of HOMUR cell lines. To confirm that these cell lines were indeed resistant to the growth arrest induced by UDCA growth curves were generated in the presence and absence of 500 micromolar UDCA for the three HOMUR lines and compared with parental HCT116 cells (Figure 2). As can be seen UDCA completely suppresses proliferation of HCT116 cells. However, all HOMUR lines were capable of growth in UDCA. It should be noted that the HOMUR17 line undergoes apoptosis spontaneously at a relatively high rate making it is difficult to obtain a growth curve that accurately represents the amount of proliferation exhibited by these cells. No such spontaneous apoptosis has ever been exhibited by either of the other HOMUR cell lines tested (data not shown). From these studies we conclude that the HOMUR cell lines are indeed resistant to UDCA-induced growth arrest.

**Figure 3:**
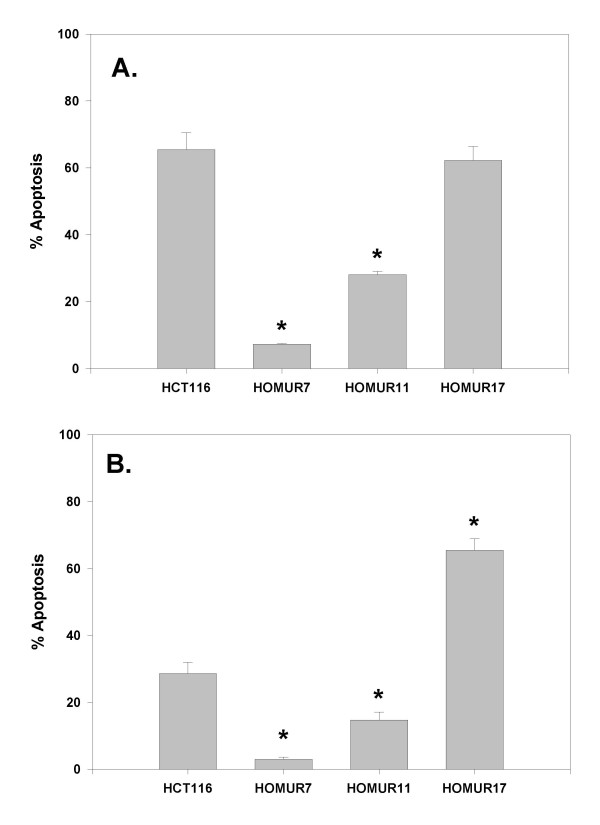
**Selected HOMUR cells show resistance to DCA-induced and hyoDCA-induced apoptosis. **HCT116, HOMUR 7, HOMUR 11, or HOMUR 17 cells were plated onto 60 mm plates and allowed to attach for 48 hours. Plates were then incubated either with (A) 500 micromolar DCA for 24 hours or (B) with 500 micromolar hyoDCA for 48 hours. Apoptosis in each culture was quantitated as described in Methods. Bars represent the average of triplicate results. Error bars represent standard deviation. Astrisks indicate a significant difference (p < 0.05) when compared with parental HCT116 cells.

We next sought to confirm that the sensitivity/resistant phenotype observed during screening of the HOMUR cell lines was reproducible. Each of the HOMUR lines were incubated with DCA or hyoDCA and apoptosis quantitated (Figure 3). As can be seen the both the HOMUR7 and HOMUR11 cell lines exhibited significantly reduced DCA and hyoDCA-induced apoptosis (p < 0.05 by t-test) relative to parental HCT116 cells. Surprisingly, HOMUR17s exhibited twice the level of hyoDCA-induced apoptosis as compared with HCT116 cells (p < 0.05 by t-test) consistent with our initial characterization of this line as being more sensitive to hyoDCA-induced apoptosis.

**Figure 5:**
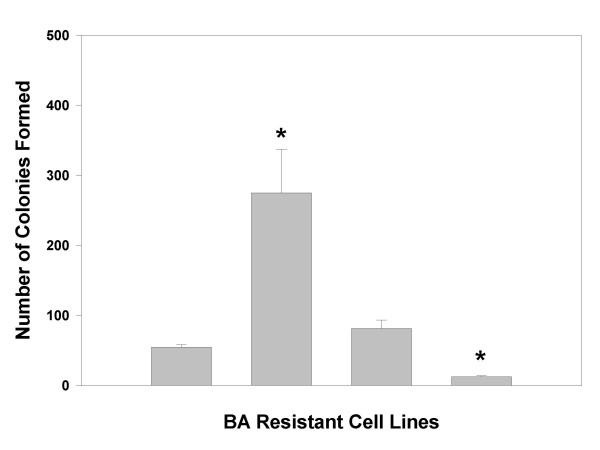
**HOMUR cells that are resistant to DCA-induced apoptosis exhibit anchorage independent growth. **HCT116, Homur7, Homur11, and Homur17 cells were plated into six well trays in 0.6% LMP agarose as described in Methods and the cells allowed to grow for 2.5 weeks. Colonies were then stained and counted. Bars depict the average number of clones per well from four independent experiments done in triplicate +/- standard deviation. Asterisks indicate a significant difference (p < 0.05) when compared with parental HCT116 cells.

We next asked whether the resistance to DCA-induced apoptosis in HOMUR lines was reflected at the biochemical level. Cleavage of poly ADP-ribose polymerase (PARP) is well known to be associated with the onset of apoptosis and so was chosen as a biochemical marker for apoptotic cell death [32]. The three HOMUR lines and HCT116 cells were incubated with 500 micromolar DCA and harvested at various times after incubation. Western blots of cell extracts were probed with anti-PARP (Figure 5). These experiments demonstrated that cleavage products of part are readily visible in HCT116 cells but are either absent (HOMUR 7 cells) or reduced (HOMUR11 cells) in apoptosis resistant cell lines. We conclude that cleavage of PARP is dramatically reduced in the most profoundly resistant HOMUR 7 cell line.

### Resistance to bile acids also confers a capacity for anchorage independent growth

We and others have previously postulated that resistance to DCA-induced apoptosis may be important in neoplastic transformation that leads to tumorigenesis in the colon [14,33]. Because the HOMUR lines exhibit resistance to bile acid-induced apoptosis we reasoned that they might be used to test this hypothesis by examining their tumorigenic properties using anchorage independent growth as a measure of their neoplastic development. Consequently, HCT116 cells and all three HOMUR lines were characterized for growth in soft agar (Figure 5). We found that the HOMUR7 line was significantly more capable of growing in soft agar than were any of the HOMUR lines or parental HCT116 cells (p < 0.05 by t-test). Notably, HOMUR17 cells which are more sensitive to DCA-induced apoptosis, showed significantly reduced soft agar growth capacity (p < 0.05 by t-test). Since anchorage independent growth was exhibited by the DCA-resistant line and not by the sensitive line we concluded that resistance to DCA-induced apoptosis correlated with anchorage independent growth.

**Figure 6:**
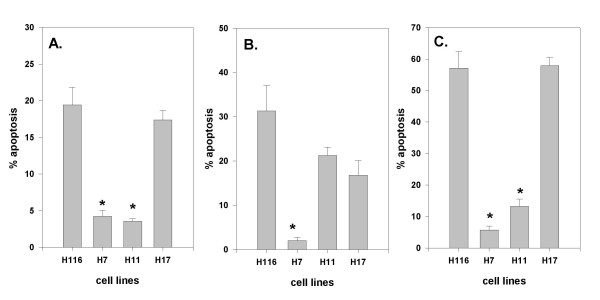
**HOMUR cells that are resistant to DCA-induced apoptosis exhibit resistance to other anti-cancer agents. **Parental HCT116 cells and HOMUR 7, 11, and 17 cells were plated at a density of 100,000 cells per 35 mm plate and incubated at 37°C for 48 hours. Subsequently etoposide (panel A) was added to a final concentration of 50 μM, cisplatin (panel B) added to a final concentration of 50 μM, or adriamycin (panel C) added to a final concentration of 1 μM to the plates. The cells were incubated with the drugs for 36 hours and then the number of apoptotic cells quantitated as described in Methods. The bars represent average values of three replicates. Error bars depict standard deviation. Astrisks denote a significant difference (p < 0.05) when compared with parental HCT116 cells.

We next asked whether resistance to bile acid-induced apoptosis could confer resistance to apoptosis induced by other agents. To examine this we tested the three HOMUR cell lines for sensitivity to apoptosis induced by three different commonly used anti-cancer agents, etoposide, cisplatin, and adriamycin. From time course analysis using HCT116 cells we determined that exposure to these agents for 36 hours was required to induce substantial apoptosis so each HOMUR cell line was exposed to the three agents for 36 hours, harvested and the fraction of apoptotic cells determined (Figure 6). As can be seen the HOMUR7 line was significantly less sensitive to all three agents (p < 0.05 by t-test). HOMUR11 cells, which are more sensitive to DCA-induced apoptosis when compared with HOMUR7s, showed significant resistance to only two of the drugs, etoposide and adriamycin. Interestingly, even the bile acid-induced apoptosis sensitive HOMUR17 cells exhibited some reduced sensitivity to cisplatin. From these experiments we conclude that resistance to bile acid-induced apoptosis can also confer resistance to other apoptosis-inducing agents.

## Discussion

In the present study we derived a set of cells that were resistant to UDCA and then tested these cells for cross resistance to the effects of two other bile acids to ascertain whether there was overlap in the signaling pathways that mediate bile acid-induced cell death. We were able to demonstrate that there is an overlap in the signaling mechanisms activated by UDCA which lead to growth arrest and those activated by DCA which bring about apoptosis. Careful examination of the number of UDCA-induced growth arrest resistant cells revealed that the majority of these lines also exhibited resistance to DCA-induced apoptosis a finding that is consistent with the concept that the signaling activities of these two bile acids may overlap. Most of the HOMUR lines exhibited some degree of resistance suggesting that the extent of overlap in signaling activities may be extensive. Hence, it seems likely that the signaling activities induced by bile acids and which are responsible for growth arrest and for apoptosis may have much in common.

The likely extensive overlap in signaling activities between DCA and UDCA raises the question of how these two bile acids can exhibit such distinctly different biological activities. Considering that all bile acids have such similar chemical structures it is not unexpected that they can also activate many of the same intracellular signaling mechanisms. However, there is also slight evidence that some bile acids interact with intracellular signaling in unique ways. For example DCA can stimulate the EGFR/ras/mek/erk signal transduction pathway, yet UDCA has been shown to suppress this same pathway [19,21]. Hence, although the same pathways may be targeted for modulation by the different bile acids the effect that they have on these pathways, activation or inhibition, may determine the ultimate biological outcome of exposure to these agents. This suggests that the distinction between tumor promotion and prevention may be very subtle

These notions emphasize the importance of elucidating the identity and the nature of the unique signaling mechanisms activated by DCA and UDCA. Insight into the characteristics of these pathways can be gleaned from our characterization of the HOMUR cells. Our observation that only the most profoundly DCA resistant HOMUR 7 line exhibits extensive growth in soft agar supports the notion that resistance to DCA-induced apoptosis favors a more tumorogenic phenotype. Our finding that HOMUR 7 cells are also markedly resistant to three commonly used anti-cancer agents suggests that DCA-induced apoptosis may utilize pathways that are also employed by cancer therapeutics. Hence, profound resistance to DCA correlates with acquired resistance to multiple other drugs each of which is known to cause cell death through very different mechanisms. Collectively these results suggest that resistance to bile acid-induced apoptosis is tumorigeneic and is consistent with findings made using natural human tumors [33].

## Conclusion

Our results strongly suggest that there is overlap in the signaling activated by DCA and UDCA. However, there is also evidence that these two bile acids may activate unique signaling pathways that may account for their diametrically opposed biological effects. Importantly, we show that resistance to DCA induced apoptosis confers a more neoplastic phenotype on tumor cells. Our results also have therapeutic implications as targeting of bile acid pathways may have unexpected consequences unless they are adequately understood.

## Competing interests

The author(s) declare that they have no competing interests.

## Authors' contributions

JDM was responsible for data analysis, drafting the manuscript and the overall direction of this study. AAP generated and partially characterized HOMUR clones. SA, SJL and RAF carried out growth curves, apoptosis assays and agarose anchorage independent growth assays. WQ carried out PARP western blots. PH assisted in characterizing HOMUR clones. All authors read and approved the manuscript.

## Pre-publication history

The pre-publication history for this paper can be accessed here:



## Figures and Tables

**Figure 4:**
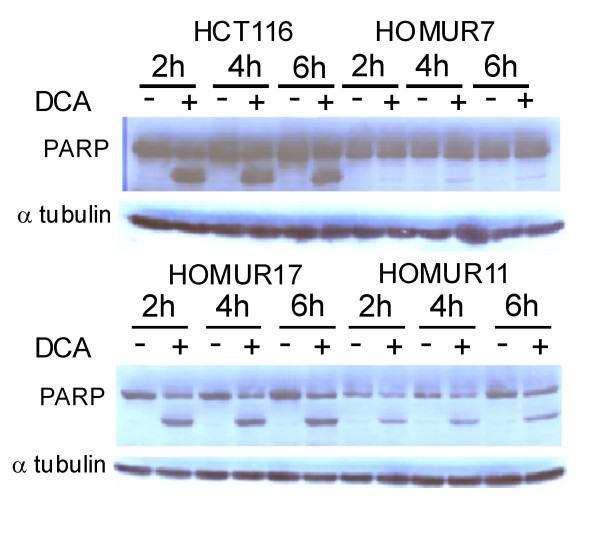
**Apoptosis resistant HOMUR cells exhibit reduced PARP cleavage when treated with DCA. **HCT116, HOMUR 7, HOMUR 11, or HOMUR 17 cells were plated onto 10 cm plates and allowed to attach for 48 hours. Cells were either untreated (-) or incubated with 500 micromolar DCA (+) and sample plates harvested at the indicated time intervals. Total cell proteins were extracted, fractionated by SDS-PAGE, and protein blots probed with antibodies against PARP. Alpha tubulin was used to control for loading variations. These experiments were repeated twice. Representative immunoblots are depicted.
